# No evidence of pathogenicity of *Dientamoeba fragilis* following detection in stools: A case-control study[Fn FN1]

**DOI:** 10.1051/parasite/2024041

**Published:** 2024-07-24

**Authors:** Germain Tchamwa Bamini, Eléna Charpentier, Emilie Guemas, Pamela Chauvin, Judith Fillaux, Alexis Valentin, Sophie Cassaing, Sandie Ménard, Antoine Berry, Xavier Iriart

**Affiliations:** 1 Parasitology-Mycology Department, Toulouse University Hospital 31059 Toulouse France; 2 Toulouse Institute for Infectious and Inflammatory Diseases (Infinity), Toulouse University, CNRS UMR5051, INSERM UMR1291, Paul Sabatier University 31024 Toulouse France; 3 RESTORE Institute, UMR 1301-Inserm 5070-CNRS EFS Paul Sabatier University 31100 Toulouse France; 4 UMR 152 PHARMA-DEV, IRD, UPS, Toulouse University 31062 Toulouse France

**Keywords:** *Dientamoeba fragilis*, Pathogenicity, Stools, *Blastocystis sp.*

## Abstract

*Dientamoeba fragilis* is a ubiquitous intestinal parasite with detection in the stools that has become increasingly frequent following the advent of PCR as a routine screening tool. However, the pathogenicity of this parasite is still much debated. In order to assess the potentially pathogenic nature of this protozoan, a retrospective case-control study was carried out between January and December 2020 on patients from Toulouse University Hospital, with the aim of evaluating the potential clinical effects and changes in laboratory parameters linked to the presence and load of *D. fragilis* in stools. After matching age, sex and mode of care (consultation or hospitalisation), no significant difference was observed in the frequency of clinical signs between the 36 patients who tested positive for *Dientamoeba fragilis* PCR in their stools and the 72 control patients who were PCR negative for this protozoan. The presence of *D. fragilis* in the faeces was not associated with changes in laboratory parameters. Furthermore, a high digestive load of *D. fragilis* had no identifiable impact on clinical and laboratory parameters. Only the concomitant presence of *Blastocystis* sp. in stools was significantly more frequent in the *D. fragilis* group (uni- and multivariate analysis). Finally, this study showed no significant difference in clinical or laboratory signs between patients carrying *Dientamoeba fragilis* and the control group, regardless of the intestinal parasite load, suggesting that *D. fragilis* could be considered a commensal of the digestive tract.

## Abstract (fr)

*Dientamoeba fragilis* est un parasite digestif ubiquitaire dont la détection dans les selles est devenue de plus en plus fréquente avec l’avènement de la PCR comme outil de détection de routine. Cependant, la pathogénicité de ce parasite est encore très discutée. Afin d’évaluer le caractère potentiellement pathogène de ce protozoaire, une étude rétrospective cas-témoins a été réalisée entre janvier et décembre 2020 sur des patients du CHU de Toulouse, dans le but d’évaluer les effets cliniques et biologiques potentiels associés à la présence et à la charge de *D. fragilis* dans les selles. Après appariement sur l’âge, le sexe et le mode de prise en charge (consultation ou hospitalisation), aucune différence significative n’a été observée dans la fréquence des signes cliniques entre les 36 patients testés positifs pour la PCR de *Dientamoeba fragilis* dans les selles et les 72 patients témoins avec une PCR négative pour ce protozoaire. La présence de *D. fragilis* dans les selles n’était pas associée à des modifications des paramètres biologiques. De plus, une charge digestive élevée de *D. fragilis* n’avait pas d’impact identifiable sur les paramètres cliniques et biologiques. Seule la présence concomitante de *Blastocystis* sp. dans les selles était significativement plus fréquente dans le groupe *D. fragilis* (analyse uni- et multivariée). En conclusion, cette étude n’a pas montré de différence significative concernant les signes cliniques ou biologiques entre les patients porteurs de *Dientamoeba fragilis* et le groupe témoin, quelle que soit la charge parasitaire digestive, indiquant que *D. fragilis* pourrait être considéré comme un commensal du tube digestif.

## Introduction

*Dientamoeba fragilis* is a ubiquitous intestinal protozoan whose prevalence varies between 0 and 62% depending on the region, population and detection method used [12]. With the development of molecular biology and the advent of PCR as a routine screening tool, the detection of *D. fragilis* in the stools has become increasingly frequent.

The pathogenicity of *Dientamoeba fragilis* is still the subject of much debate in the literature [4] and the cause-and-effect relationship between *D. fragilis* and gastrointestinal symptoms has yet to be established. These discordant results regarding the pathogenicity of *D. fragilis* cannot be explained by *D. fragilis* genotype variation since there is a strong predominance of genotype 1 in both humans and a few animal hosts [7]. Recent studies have shown that a very low level of genetic variability characterises parasite isolates collected in various geographical areas and from both symptomatic and asymptomatic cases [6]. Another hypothesis to explain these discrepancies could be linked to the digestive *D. fragilis* load. In the same way as *Candida albicans*, which is a commensal yeast of the digestive tract that can become pathogenic when its density increases [22], a similar phenomenon cannot be excluded for *D. fragilis*. A low digestive load of *D. fragilis* could therefore be completely asymptomatic, with digestive symptoms only appearing when the parasite load becomes higher.

Here, a retrospective case-control study was carried out to evaluate the potential clinical effects and changes in laboratory parameters associated with the presence and load of *Dientamoeba fragilis* in the stools, to assess the potentially pathogenic nature of this parasite.

## Materials and methods

### Ethics statement

All the faeces samples were collected from patients who had undergone a parasitological stool examination by PCR. Samples were obtained only for routine diagnosis on the basis of physician prescriptions. Clinical data were anonymised for analysis. According to French Public Health Law [9], this protocol did not require Ethics Committee approval and was exempt from the requirements of formal informed consent.

### Study design

A retrospective study was performed on all the patients from Toulouse University Hospital who underwent a PCR assay targeting *Dientamoeba fragilis* in the stools between January and December 2020 inclusive. This *D. fragilis* PCR was routinely carried out on all patients for whom a parasitological stool examination was prescribed. Each patient who tested positive for *D. fragilis* PCR was paired to two control patients with a negative diagnosis for *D. fragilis*. Patients were matched on age, sex and mode of care (consultation or hospitalisation). Demographic, clinical and laboratory data were assessed between the 2 groups.

To assess the effect of *Dientamoeba fragilis* load on these same parameters, a second analysis was carried out to compare patients with the highest digestive loads of *D. fragilis* and control patients. Only patients with a Ct < 28 were included in this second analysis.

### Routine stool DNA extraction and multiplex real-time PCR

For each patient, a 250–500 mg stool sample (or 250 μL for liquid stool) was suspended in 1 mL of phosphate-buffered saline (PBS) and homogenised by bead beating at 7000 rpm for 70 s (MagNA Lyser, Roche Diagnostics, Mannheim, Germany). DNA extraction was performed using a High Pure PCR Template Preparation Kit (Roche Diagnostics, Meylan, France), according to the manufacturer’s instructions. Briefly, 200 μL of binding buffer and 50 μL proteinase K were added to 200 μL of stool suspension. After 10-min incubation at 70 °C, 100 μL of isopropanol was added and the solution was centrifuged through a filter tube for 1 min at 8000×*g*. The filter tube was subsequently centrifuged for 1 min at 8000×*g* after adding 500 μL of inhibitor removal buffer and washed three times with wash buffer. The DNA was then eluted in 200 μL of elution buffer by centrifuging for 1 min at 8000×*g*. Routine molecular detection of *Dientamoeba fragilis* was conducted using an Amplidiag® Stool Parasites Real-Time PCR Kit (Hologic formerly Mobidiag, Espoo, Finland), according to the manufacturer’s recommendations. A PCR cycle threshold value (Ct) was obtained for all patients who tested positive for *D. fragilis*, enabling semi-quantification of the parasite load. The Amplidiag® Stool Parasites Real-Time PCR Kit also detected *Giardia intestinalis*, *Cryptosporidium* spp. and *Entamoeba histolytica.* This assay comes with a calibration kit for Amplidiag® Stool Parasites (AD-SPC-30) and each run includes positive and negative controls. Detection of viral and bacterial pathogens from stool samples was performed using the Amplidiag® Bacterial GE (*Campylobacter jejuni* and *C. coli, Salmonella*, *Shigella, Yersinia* and diarrheagenic *E. coli* species) and the Amplidiag® Viral GE multiplex assays (*Rotavirus, Norovirus, Sapovirus, Adenovirus, Astrovirus*) (Hologic formerly Mobidiag, Espoo, Finland), according to the manufacturer’s recommendations. After PCR amplification with a CFX96 instrument (Bio-Rad, Richmond, VA, USA), data were analysed with Amplidiag® Analyzer software (Hologic formerly Mobidiag, Espoo, Finland) using internal thresholds.

### Routine microscopy for intestinal parasites

All the stool samples from this study were examined by microscopic methods to detect intestinal helminth (such as *Enterobius vermicularis*) or protozoa not targeted by PCR (such as *Blastocystis* sp*., Cystoisospora belli, Cyclospora cayetanensis, Balantioides coli*). Routine microscopic examination was performed upon receipt of the sample by experienced microscopists after staining with merthiolate-iodine-formaldehyde colouration [34] and concentration using the Ovatec® Plus flotation technique (Zoetis) and Bailenger method (Para-selles®, Biosynex®, Illkirch-Graffenstaden, France) according to the manufacturer’s recommendations.

### Analysed data

For both cases and controls, demographic data were collected (age, sex, residence, travels, countries visited, countries of origin, reason for prescription and mode of care) as well as clinical data, including the presence of digestive or systemic symptoms (diarrhoea, abdominal pain, constipation, nausea or vomiting, anorexia, weight loss, fever and asthenia), the notion of immunosuppression or risk factors (HIV, transplantation, other immunosuppression) and use of a treatment by imidazole or anti-parasitic drugs. Some laboratory parameter data were also compiled (microbiological examination of stools, cell blood count, C-reactive protein).

### Statistical methods

Data were analysed with SIGMA Stat1 software (2.03; Jandel Corporation, San Jose, CA, USA).

Values were reported as the median and interquartile range (IQR) [25%; 75%]. For a two-group comparison, data were compared using the Mann–Whitney Rank Sum test. Patient characteristics in each group were compared using the Chi-Square test or Fisher’s Exact test as appropriate. Absolute differences detectable with the sample size of this matched case-control study were calculated (power = 80%; *α* = 5%) with the R package epiR tool (R package version 0.9-69). Variables with *p*-value ≤ 0.10 by univariate analysis as well as variables associated with the initial hypothesis were incorporated in the multivariate analysis (logistic or multiple linear regression). Relative risks were obtained by odds ratios (OR) with 95% confidence intervals (95% CI). A comparison was considered statistically significant if the *p*-value was ≤0.05.

## Results

### Characteristics of patients positive or negative for *Dientamoeba fragilis*

**Figure 1:**
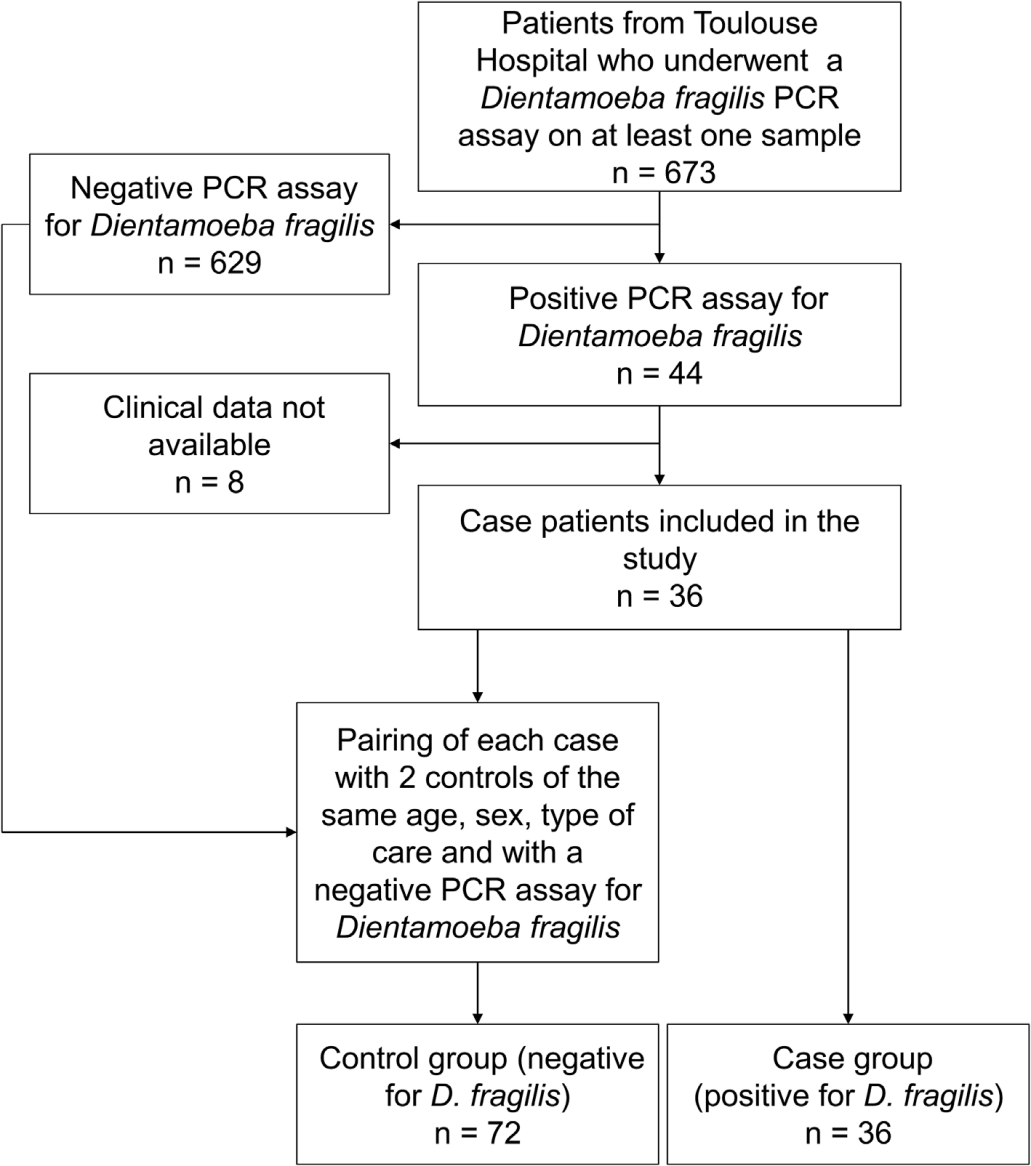
Flow chart of patient inclusion.

Between January and December 2020, 673 patients from Toulouse University Hospital underwent a PCR assay targeting *Dientamoeba fragilis* on at least one stool sample. Among these patients, 44 (6.5%) had a positive PCR result for *D. fragilis* (Fig. 1).

Demographic characteristics of patients testing positive or negative for *D. fragilis* detection are outlined in Table 1. There was a non-significant trend towards a lower proportion of female patients in the uninfected group (39.1%) compared with the *D. fragilis* group (47.7%) (*p* = 0.26, Chi^2^). The median age of *D. fragilis* stool-positive patients was 35.4 [13.9; 59.7] with an over-representation of younger patients (particularly those under 15 years of age) (Table 1). In terms of age, the epidemiology of patients with *D. fragilis* was therefore very different from that of other patients usually managed at the Toulouse Hospital Laboratory and who are predominantly much older.

**Table 1:** Baseline characteristics of patients with or without *Dientamoeba fragilis.*

	Patients with *D. fragilis*	Patients without *D. fragilis*	*p*-value
Total, *n*	44	629	
Male/female patients, *n*	(23/21)	(383/246)	0.26^a^
Age, years (median)	35.4 [13.9; 59.7]	56.6 [39.2; 66.5]	**<0.001** ^**b**^
Age classes, *n* (%)			
0–15	12 (27.3%)	45 (7.2%)	
15–30	7 (15.9%)	28 (10.2%)	
30–45	5 (11.4%)	90 (14.3%)	**<0.001** ^**a**^
45–60	9 (20.5%)	165 (26.2%)	
>60	11 (25%)	265 (42.1%)	

^a^Calculated by Chi-Square test; ^b^Calculated by Mann–Whitney Rank Sum test.

*D. fragilis: Dientamoeba fragilis.*

Bold indicates a *p*-value < 0.05.

### Clinical and laboratory factors associated with the detection of *Dientamoeba fragilis* in the stools (uni- and multivariate analysis)

A case-control study was performed to evaluate the potential clinical and laboratory effects associated with the presence of *Dientamoeba fragilis* in the stools. Of the 44 patients with a positive PCR result for *D. fragilis* in the stools, 36 patients had an available and complete clinical file and were included in the case-control study. These 36 patients were paired with two controls each (72 controls) on three variables: age, sex and the mode of care (consultation or hospitalisation). The difference in age and sex-ratio observed in the baseline characteristics of patients with or without *D. fragilis* justified matching for these parameters. Matching on the variable “consultation /hospitalisation” made it possible to avoid bias due to varying levels of severity when patients are taken into care.

After completion of matching on age, sex and mode of care, characteristics of the 36 cases patients with *Dientamoeba fragilis* were compared with the 72 control patients without *D. fragilis* (Table 2). With this sample size, this matched case-control study was able to detect an absolute difference varying from 17.8 to 28.4%, for an exposure in control patients varying from 5 to 40% (power = 80%; *α* = 5%).

**Table 2:** Epidemiological and clinical characteristics of the case and control populations after matching according to sex, age and type of care.

	Cases: patients with *D. fragilis*	Controls: patients without *D. fragilis*	*p*-value (1)	Patients with high load of *D. fragilis* (Ct < 28)	*p*-value (2)
Total, *n*	36	72		20	
Male/female patients, *n*	(21/15)	(42/30)	1.00^a^	(12/8)	0.89^a^
Age, years, med IQR [25%; 75%]	30.4 [14.8; 58.2]	29.1 [16.8; 59.1]	0.90^b^	45.1 [14.9; 58.2]	0.90^b^
Medical care, *n* (%)					
Consultation	14 (38.9%)	28 (38.9%)	1.00^a^	9 (45.0%)	0.62^a^
Hospitalisation	22 (61.1%)	44 (61.1%)		11 (55.0%)	
Residence, *n* (%)					
Town	31 (86.1%)	62 (86.1%)	1.00^a^	17 (85.0%)	1.00^a^
Rural	5 (13.9%)	10 (13.9%)		3 (15.0%)	
Travel in the previous 2 years, *n* (%)					
Outside metropolitan France	7 (22.6%)	18 (25.0%)	0.79^a^	4 (25.0%)	1.00^c^
Outside Europe	7 (22.6%)	13 (18.1%)	0.59^a^	4 (25.0%)	0.50^c^
Migrant, *n* (%)	3 (8.3%)	9 (12.5%)	0.52^a^	3 (15.0%)	0.72^c^
Immunosuppression	5 (13. 9%)	20 (27.8%)	0.16^a^	2 (10.0%)	0.34^c^
Cancer	1 (2.8%)	8 (11.1%)	0.14^a^	1 (5.0%)	0.68^c^
Graft or transplantation	2 (5.6%)	7 (9.7%)	0.46^a^	1 (5.0%)	0.68^c^
HIV	0 (0.0%)	1 (1.4%)	0.48^a^	0 (0.0%)	1.00^c^
Systemic disease	2 (5.6%)	4 (5.6%)	1.00^a^	1 (5.0%)	1.00^c^
Comorbidity, *n* (%)					
Diabetes mellitus	3 (8.3%)	8 (11.1%)	0.65^a^	2 (10.0%)	1.00^c^
Cardiovascular risks	5 (13.9%)	6 (8.3%)	0.37^a^	3 (15.0%)	0.40^c^
Other risk factors^d^	3 (8.3%)	7 (9.7%)	0.81^a^	3 (15.0%)	0.45^c^
Digestive symptoms, *n* (%)					
At least 1 digestive symptom	18 (50%)	48 (66.7%)	0.09^a^	10 (50.0%)	0.17^a^
Diarrhoea	8 (22.2%)	26 (36.1%)	0.14^a^	4 (20.0%)	0.17^a^
Abdominal pain	14 (38.9%)	26 (36.1%)	0.78^a^	8 (40.0%)	0.75^a^
Constipation	2 (5.6%)	8 (11.1%)	0.35^a^	1 (5.0%)	0.68^a^
Nausea/vomiting	6 (16.7%)	7 (9.9%)	0.31^a^	4 (20.0%)	0.25^c^
General signs, *n* (%)					
At least 1 general symptom	8 (22.9%)	24 (33.8%)	0.25^a^	4 (20.0%)	0.24^a^
Anorexia	2 (5.6%)	5 (6.9%)	0.78^a^	1 (5.0%)	1.00^c^
Weight loss	3 (8.6%)	10 (14.1%)	0.42^a^	2 (10.0%)	1.00^a^
Fever	4 (11.1%)	18 (25%)	0.09^a^	2 (10.0%)	0.22^c^
Asthenia	5 (14.3%)	10 (14.1%)	0.98^a^	2 (10.0%)	1.00^c^

^a^Calculated by Chi-Square test; ^b^Calculated by Mann–Whitney Rank Sum test; ^c^Calculated by Fisher’s Exact test; ^d^Including cirrhosis, liver fibrosis, renal failure, heart failure.

(1): statistical analysis between cases and controls.

(2): statistical analysis between patients with high load and controls.

med IQR [25%; 75%]: median interquartile range [25%; 75%].

*D. fragilis: Dientamoeba fragilis.*

Bold indicates a *p*-value < 0.05.

Between the case and control groups, there were no significant differences in terms of residence, travel and migration. Comparison of comorbidity factors showed no significant difference between the 2 groups for diabetes, cardiovascular risk factors or other comorbidity factors. Similarly, no significant difference was observed between the case and control groups with regard to the existence of immunosuppression (solid organ transplantation, chemotherapy, haematopoietic stem cell transplantation, cancer, HIV, systemic disease and immunosuppressive treatment).

There was a non-significant trend towards a lower proportion of patients exhibiting digestive symptoms (50%) in the case group than in the control group (66.7%) (*p* = 0.09, Chi^2^), mainly due to a lower proportion (also non-significant) of patients presenting with diarrhoea in the *Dientamoeba fragilis* group (22.2% vs 36.1%; *p* = 0.14, Chi^2^). Among the digestive symptoms collected, the manifestations most frequently encountered in the group of patients with *D. fragilis* were abdominal pain (38.9%), diarrhoea (22.2%) and nausea/vomiting (16.7%). However, there was no significant difference between the 2 groups for any of the clinical symptoms. With regard to systemic signs (anorexia, fever, asthenia and weight loss), no significant differences were observed between the two groups, but there was a non-significant trend towards fewer patients experiencing fever (11.1%) in the *D. fragilis* group than in the control group (25%) (*p* = 0.09, Chi^2^).

Laboratory characteristics and treatment of the 36 cases and 72 control patients are outlined in Table 3. Within the biological blood parameters collected, there were no significant differences between the two groups with the exception of C-reactive protein, which tended to be lower in the case group than in the control group (*p* = 0.10, Mann–Whitney Rank Sum test).

**Table 3:** Laboratory characteristics and treatment of the case and control populations after matching according to sex, age and type of care.

	Cases: patients with *D. fragilis*	Controls: patients without *D. fragilis*	*p*-value (1)	Patients with high load of *D. fragilis* (Ct < 28)	*p*-value (2)
Total, *n*	36	72		20	
Blood parameters, med IQR [25%; 75%]					
Red blood cells (T/L)	4.5 [4.2; 4.9]	4.5 [3.9; 5.0]	0.42^b^	4.6 [4.5; 5.3]	0.12^b^
Haemoglobin (g/dL)	13.4 [12.3; 14.6]	12.6 [11.5; 14.2]	0.20^b^	14.1 [13.4; 14.9]	**0.01** ^**b**^
Total leucocytes (G/L)	7.4 [5.9; 10.6]	7.5 [5.2; 9.77]	0.36^b^	6.7 [5.9; 9.0]	0.92^b^
Polymorphonuclear neutrophils (G/L)	3.5 [2.6; 5.8]	4.8 [3.1; 5.9]	0.52^b^	3.2 [2.6; 4.3]	0.13^b^
Polymorphonuclear eosinophils (G/L)	0.3 [0.1; 1.2]	0.1 [0.1; 0.4]	0.16^b^	0.35 [0.1; 1.2]	**0.05** ^**b**^
Polymorphonuclear basophils (G/L)	0.00 [0.00; 0.1]	0.00 [0.00; 0.1]	0.94^b^	0.05 [0.00; 0.1]	0.63^b^
Lymphocytes (G/L)	2.2 [1.4; 3.0]	2.1 [1.2; 2.8]	0.59^b^	1.9 [1.5; 2.9]	0.87^b^
Mononuclear leucocytes (G/L)	0.6 [0.5; 0.7]	0.5 [0.4; 0.8]	0.84^b^	0.5 [0.4; 0.6]	0.43^b^
Blood platelets (G/L)	220 [170; 288]	262 [194; 347]	0.17^b^	206 [163; 278]	0.11^b^
C-reactive protein (mg/L)	0.6 [0.6; 8.6]	3 [0.7; 36.5]	0.10^b^	0.6 [0.6; 2.6]	**0.02** ^**b**^
Concomitant infections, *n* (%)					
Other protozoa in the stools	8 (22.2%)	4 (5.6%)	**<0.01** ^**a**^	4 (20.0%)	0.07^c^
*Blastocystis* sp.	7 (20.0%)	1 (1.4%)	**<0.001** ^**a**^	3 (15.0%)	**0.03** ^**c**^
Helminths	5 (14.3%)	9 (12.5%)	0.80^a^	3 (15.0%)	0.72^c^
*Enterobius vermicularis*	1 (2.9%)	0 (0.0%)	0.15^a^	1 (5.0%)	0.22^c^
Viral co-infections	3 (8.6%)	7 (9.7%)	0.85^a^	2 (10.0%)	1.00^c^
Bacterial co-infections	6 (17.7%)	14 (19.4%)	0.83^a^	4 (20.0%)	1.00^c^
Treatments before *D. fragilis* PCR, *n* (%)					
Imidazoles	2 (6.1%)	2 (2.8%)	0.42^a^	1 (5.3%)	0.51^c^
Other anti-parasitic drugs^d^	1 (3.0%)	2 (2.8%)	0.95^a^	0 (0.0%)	1.00^c^
Treatments after *D. fragilis* PCR, *n* (%)					
Imidazoles	9 (27.3%)	8 (11.3%)	**0.04** ^**a**^	3 (15.8%)	0.69^c^
Other anti-parasitic drugs^d^	11 (33.3%)	20 (28.2%)	0.59^a^	9 (47.4%)	0.11^a^
Antibiotics	4 (12.9%)	14 (19.7%)	0.41^a^	0 (0.0%)	0.06^c^
Anti-spasmodic drugs	3 (9.4%)	6 (8.5%)	0.88^a^	2 (11.1%)	0.66^c^
Anti-diarrhoeal drugs	1 (3.1%)	2 (2.8%)	0.93^a^	0 (0.0%)	1.00^c^

^a^Calculated by Chi-Square test; ^b^Calculated by Mann–Whitney Rank Sum test; ^c^Calculated by Fisher’s Exact test; ^d^Including flubendazole, albendazole, ivermectin, praziquantel, niclosamide.

(1): statistical analysis between cases and controls.

(2): statistical analysis between patients with high load and controls.

med IQR [25%; 75%]: median interquartile range [25%; 75%].

*D. fragilis: Dientamoeba fragilis.*

Bold indicates a *p*-value < 0.05.

Among patients infected with *Dientamoeba fragilis*, eight patients (22.2%) had another protozoan in their faeces, whereas only four patients (5.6%) were co-infected in the control group (*p* < 0.01, Chi^2^). This difference between the two groups was essentially due to the concomitant presence of *Blastocystis* sp. (20% in the *D. fragilis* group *versus* 1.4% in the control group) (*p* < 0.001, Chi^2^). *Enterobius vermicularis* (pinworm) was detected only once in the study (*D. fragilis* group) with no significant difference compared with the control group (*p* = 0.15, Chi^2^). There were no significant differences between the groups in terms of other parasitic, viral or bacterial infectious aetiologies.

In the month before the realisation of the *Dientamoeba fragilis* PCR assay, there was no significant difference in the proportion of patients who received treatment with imidazoles or other anti-parasitic drugs. However, analysis of the various treatments prescribed in the 30 days following the *D. fragilis* PCR test (imidazole, anti-parasitics, antibiotics, anti-spasmodics and anti-diarrhoeal drugs) showed a significantly higher prescription of imidazole in the case group (27.3%) than in the control group (11.3%) (*p* = 0.04, Chi^2^). One hypothesis to explain this over-representation of imidazole prescriptions in the case group is the possible intention of clinicians to treat patients detected positive for *D. fragilis* or *Blastocystis* sp. (which is more frequent in the case group). In fact, this drug is traditionally prescribed for these two indications [2, 27].

Variables with *p*-value ≤ 0.10 by univariate analysis as well as the variables associated with the initial hypothesis were incorporated in the multivariate analysis (multiple logistic regression). The variable “treatment with imidazole after realisation of PCR *Dientamoeba fragilis”* was not included in the analysis as the difference between the 2 groups was probably due to the discovery of *D. fragilis* and not an explanatory cause.

Of the parameters analysed in the multivariate study, only the concomitant presence of *Blastocystis* sp. in the stools remained significant in the multivariate analysis (odds ratio, OR [95% CI]: 35.8 [1.9; 672.9], *p* = 0.02) (Table 4).

**Table 4:** Multivariate analysis (Multiple logistic regression).

Variables	Odds ratio	OR [95% CI]	*p*-value
*Blastocystis* sp. in the stools	**35.8**	**1.9–672.9**	**0.02**
Digestive symptoms	0.42	0.11**–**1.58	0.20
Fever	1.11	0.20**–**6.07	0.91
C-reactive protein concentration	0.99	0.97**–**1.01	0.17

Bold indicates a *p*-value < 0.05.

### Clinical and laboratory factors associated with the detection of a high load of *Dientamoeba fragilis* in the stools (uni- and multivariate analysis)

In order to assess the impact of the digestive load of *Dientamoeba fragilis* on the various parameters studied, case group patients with a high *D. fragilis* parasite load (defined by a PCR cycle threshold value (Ct) < 28) were compared with control patients (Tables 2 and 3).

Comparison of the various parameters between these 20 patients with a high *Dientamoeba fragilis* load and control patients did not reveal any significant difference, except for the presence of *Blastocystis* sp. in the faeces (*p* = 0.03, Fisher’s Exact test), haemoglobin concentration (*p* = 0.01, Mann–Whitney Rank Sum test), polymorphonuclear eosinophil level (*p* = 0.05, Mann–Whitney Rank Sum test) and C-reactive protein concentration (*p* = 0.02, Mann–Whitney Rank Sum test) (Table 3). Patients with a high *D. fragilis* parasite load did not exhibit significantly more digestive symptoms than control patients (Table 2).

In the multivariate analysis, the considered parameters were variables that were significant in the univariate analysis (*Blastocystis* sp. in the faeces, haemoglobin concentration, polymorphonuclear eosinophil level and C-reactive protein concentration) and the most significant variables associated with our initial hypothesis (digestive symptoms). Of the parameters analysed, only the concomitant presence of *Blastocystis* sp. in the stools remained statistically significant (*p* = 0.04, multiple linear regression) (Table 5).

**Table 5:** Multivariate analysis (Multiple linear regression) according to *Dientamoeba fragilis* parasite load.

Variables	*p*-value
*Blastocystis* sp. in the stools	**0.04**
Haemoglobin concentration	0.66
Polymorphonuclear eosinophil level	0.41
C-reactive protein concentration	0.42
Digestive symptoms	0.31

Bold indicates a *p*-value < 0.05.

## Discussion

*Dientamoeba fragilis* is a cosmopolitan protozoan. Despite its high prevalence, our knowledge of this protozoan remains incomplete, particularly with regard to its transmission and pathogenicity. While most case-control studies have compared the frequency of *D. fragilis* detection in symptomatic or non-symptomatic individuals, we opted instead for a comparison of clinical and laboratory parameters between patients with and without *D. fragilis* in the stools. This methodology enabled us not only to observe the relationship between the presence of *D. fragilis* and the incidence of digestive symptoms, but also to assess its impact on other parameters (laboratory parameters, systemic clinical signs and epidemiological data).

In our study, there was no significant difference in the frequency of clinical signs of any kind between patients with a positive PCR assay for *Dientamoeba fragilis* and control groups after completion of matching according to age, sex and type of care. Furthermore, we did not identify any links between a high load of *D. fragilis* in the faeces and the presence of more frequent clinical symptoms compared with the control patients.

The existence of a causal relationship between the presence of digestive symptoms and detection of *Dientamoeba fragilis* in the stools remains fairly controversial in the literature. While *D. fragilis* was initially described as non-pathogenic by Jepps and Dobell [18], numerous studies have reported possible pathogenicity either by correlating gastrointestinal symptoms with the presence of this parasite [37, 39] or by observing an improvement in symptoms after administration of an anti-protozoan drug [23, 33]. However, the conclusions of these studies are sometimes questionable since they are based on indirect results with no proven causal links. In addition, numerous studies have shown *D. fragilis* carriage without any clinical signs [30].

Case-control studies evaluating the prevalence of *Dientamoeba fragilis* infection in symptomatic or non-symptomatic individuals are also available in the literature. Of these case-control studies, a very small number identified potential pathogenicity of *D. fragilis* in association with more frequent gastrointestinal symptoms or diarrhoea in groups carrying the protozoan [2, 28]. The vast majority of comparative studies using *D. fragilis* PCR assays as a diagnostic method demonstrated no statistically significant difference in terms of the frequency of gastrointestinal signs between patients with the parasite in their stools and negative control subjects [14, 16, 17, 19, 20, 25, 26, 31], or even a higher incidence of *D. fragilis* detection in asymptomatic patients than in symptomatic ones [3–5]. Very recently, in a large study that included nearly 28,000 patients, Shasha et al. showed a significantly lower occurrence of digestive symptoms [adjusted OR 0.82 (0.76–0.88)] in patients testing positive for *D. fragilis* on PCR assay compared to control patients negative for this parasite [36]. Consistently with our results, all these studies reveal no evidence of pathogenicity of this parasite and tend to suggest that *D. fragilis* is not pathogenic.

One of the strengths of our study was the analysis of the impact of *Dientamoeba fragilis* detection in the stools on clinical and laboratory parameters according to protozoan load in the stools. In the study carried out by Shasha et al., higher parasitic burdens were not linked to a greater incidence of digestive symptoms [36]. In two other case control studies, digestive *D. fragilis* loads (evaluated by Ct value) were even greater in asymptomatic patients than in patients with intestinal symptoms [3, 5]. These studies, like our own, did not reveal any clinical signs that were more frequently associated with a high *D. fragilis* load. Even though these results are based on evaluation of the Ct value and have some limitations inherent to this method of semi-quantification (homogeneity of stools, PCR inhibitor, sampling), they contradict the idea that the pathogenicity of these parasites is load dependent.

For a long time, the impact of immune deficiency on *Dientamoeba fragilis* infection and disease has remained unclear. In short, *D. fragilis* is sometimes detected in immunocompromised patients [10, 21] but Sarzhanov et al. demonstrated that the prevalence of *D. fragilis* detection in the stools did not differ statistically between immunocompromised and immunocompetent patients [35]. Our data were consistent with these results and immune deficiencies appear to be low impact on *D. fragilis* frequency of detection.

Some authors have described *Dientamoeba fragilis* as a potential cause of travellers’ diarrhoea due to the presence of the parasite in returning travellers [38]. Nevertheless, the study by van Hattem et al. revealed that a substantial proportion of travellers from high-income countries were *D. fragilis* carriers before their trips [15]. As in the study conducted by Gefen-Halevi et al. [11], our results did not highlight a link between the detection of *D. fragilis* and travel.

In the literature, the presence of *Dientamoeba fragilis* in the stools has rarely been correlated with changes in laboratory parameters. A few recent studies have evaluated the concentration of faecal calprotectin (a marker of inflammation in the digestive tract) in patients infected with *D. fragilis*, but results are contradictory [1, 4]. The Brands *et al.* study indicated no difference in faecal calprotectin concentration between two groups of patients (with or without *D. fragilis* in the stools) regardless of the presence of digestive symptoms [4]. Conversely, Aykur *et al.* detected a higher level of faecal calprotectin in a group of symptomatic patients with *D. fragilis* compared to healthy or symptomatic patients without the protozoan [1]. In the analysis including all the positive *D. fragilis* patients of our study, there was no significant difference among the biological blood parameters collected between the two groups. Haemoglobin levels were higher for patients with a high digestive load of *D. fragilis*, while C-reactive protein concentration was lower in patients carrying *D. fragilis* in the univariate analysis (but not in multivariate analysis). For these two parameters, these unexpected association could be related to the better general health of patients carrying *D. fragilis*. These results tally with numerous studies that found more prevalent *D. fragilis* digestive carriage in asymptomatic or non-immunocompromised patients [3, 5, 35].

In our study, polymorphonuclear eosinophil level was greater in patients with a high digestive load of *Dientamoeba fragilis* (only in the univariate analysis). Eosinophilia has already been described in relation to the presence of *D. fragilis* in the stools [13]. It is difficult to know whether eosinophilia is directly connected to the presence of *D. fragilis* or whether it may be due to a concomitant helminthic infection. Correlations between *D. fragilis* and *Enterobius vermicularis* (pinworm) have often been described in the literature [8]. Many authors have suggested that *D. fragilis* is transmitted *via Enterobius* eggs [29, 32]. However, only one patient from the *D. fragilis* group as part of our study had pinworm in his stools (non-significant difference between the two groups), but the diagnosis of enterobiasis may be underestimated in our study due to detection based only on stool microscopic examination after concentration (Scotch Tape test not carried out).

Conversely, the presence of a digestive co-infection with *Blastocystis* sp. was significantly more frequent in patients infected with *Dientamoeba* (uni- and multivariate analysis), even when only high *D. fragilis* loads were considered. *Blastocystis* sp. was found in 20% of patients infected with *D. fragilis*, whereas it was observed only once in the control groups (1.4%). This high prevalence of *D. fragilis/Blastocystis* sp. co-detection in the stools has already been described in the literature [14, 24]. It would appear that the clinical significance of the digestive presence of *D. fragilis* and *Blastocystis* sp. is identical for the two parasites. Importantly, the incidence of *D. fragilis* and *Blastocystis* sp. was lower in cases exhibiting gastroenteric symptoms than in control groups, as demonstrated in a study by de Boer et al. [3]. Furthermore, no link could be made between the existence of gastrointestinal symptoms and the presence of these two parasites [36]. According to the authors, this data suggests that the presence of these protozoa is considered to be related to a healthy intestinal microbiota [3, 36].

The results and interpretations of our study are limited by its retrospective design, and data may be incomplete in terms of the information obtained. Another limitation of the study is the relatively limited number of samples, which does not allow detection of a small absolute difference. Nevertheless, no statistical trend concerning the potential pathogenicity of *Dientamoeba fragilis* (presence of digestive or systemic clinical signs, biology), was associated with the presence of this parasite in this study. Conversely, patients carrying *D. fragilis* seemed to have better general health for most of these parameters, which is entirely consistent with larger-scale studies on *D. fragilis* [36]. It is therefore highly unlikely that an increase in the number of patients included in the study would have modified these results. Moreover, using the *Dientamoeba fragilis* PCR assay as an inclusion criterion could induce a selection bias related to the absence of healthy subjects. Nevertheless, this strategy enabled us to observe how the presence of *D. fragilis* is associated with a very wide range of parameters other than digestive symptoms (laboratory parameters, systemic clinical signs, epidemiological data).

## Conclusion

The development of PCR-based diagnostic techniques has led to increased number of patients being detected positive for *Dientamoeba fragilis*. The controversial pathogenicity of this intestinal parasite and an apparent uptick in the incidence of positive cases have reignited discussions between clinicians and microbiologists on how best to consider a positive patient. As observed in several recent studies, our study showed no significant difference in clinical or laboratory signs between patients carrying *D. fragilis* and the control group, regardless of the digestive parasite load. Our work therefore supports the idea that *D. fragilis* should probably not be considered a pathogenic protozoan, but rather a commensal of the digestive tract. Routine diagnosis of this protozoan in the stools and anti-*Dientamoeba* treatment therefore does not necessarily appear to be useful.
